# Association between Acute Respiratory Distress Syndrome Due to COVID-19 and Long-Term Sleep and Circadian Sleep–Wake Disorders

**DOI:** 10.3390/jcm12206639

**Published:** 2023-10-20

**Authors:** Mario Henríquez-Beltrán, Iván Benítez, Thalía Belmonte, Jorge Jorquera, Jorge Jorquera-Diaz, Igor Cigarroa, Matías Burgos, Rocio Sanhueza, Claudia Jeria, Isabel Fernandez-Bussy, Estefania Nova-Lamperti, Ferrán Barbé, Adriano Targa, Gonzalo Labarca

**Affiliations:** 1 Núcleo de Investigación en Ciencias de la Salud, Universidad Adventista de Chile, Chillán 3780000, Chile; eliashbm@hotmail.com; 2 Translational Research in Respiratory Medicine, Hospital Universitari Arnau de Vilanova-Santa Maria, Biomedical Research Institute of Lleida (IRBLleida), 25198 Lleida, Spain; ibenitez@irblleida.cat (I.B.); thaliabelmonte@gmail.com (T.B.); febarbe.lleida.ics@gencat.cat (F.B.); adrianotargads@gmail.com (A.T.); 3 CIBER of Respiratory Diseases (CIBERES), Institute of Health Carlos III, 28029 Madrid, Spain; 4 Centro de Enfermedades Respiratorias, Clínica Las Condes, Facultad de Medicina, Universidad Finis Terrae, Santiago 7591047, Chile; jjorquera@clc.cl; 5 Facultad de Ciencias Medicas, Universidad Favarolo, Buenos Aires C1079ABE, Argentina; jorgejorquera19@gmail.com; 6 Escuela de Kinesiología, Facultad de Salud, Universidad Santo Tomás, Santiago 8370003, Chile; icigarroa@santotomas.cl (I.C.); m.burgos.n12@gmail.com (M.B.); rociosanhuezaroa8@gmail.com (R.S.); 7 Área Transversal de Formación General, Unidad de Idiomas, Universidad Santo Tomás, Santiago 8370003, Chile; claudiajeriafe@santotomas.cl; 8 Departamento de Medicina, Facultad de Ciencias Medicas, Universidad Católica Argentina, Buenos Aires C1107AFB, Argentina; izzyfb@gmail.com; 9 Laboratorio de Inmunología Molecular y Traslacional, Departamento de Bioquímica Clínica e Inmunología, Facultad de Farmacia, Universidad de Concepción, Concepción 4070112, Chile; enova@udec.cl; 10 Department of Clinical Biochemistry and Immunology, Faculty of Pharmacy, University of Concepción, Concepción 4070112, Chile; 11 Division of Sleep Medicine, Beth Israel Deaconess Medical Center, Harvard Medical School, 330 Brookline Ave., Boston, MA 02215, USA

**Keywords:** sleep, circadian rest–activity rhythm, ARDS, sleep apnea, COMISA

## Abstract

Current studies agree on the impact of sleep and circadian rest–activity rhythm alterations in acute respiratory distress syndrome (ARDS) survivors. However, research on the duration of this impact is scarce. In this study, we evaluate the impact of ARDS on the sleep and circadian rest–activity rhythm of COVID-19 survivors twelve months after hospital discharge. This is a prospective study including COVID-19 survivors with and without ARDS during hospitalization. Data was collected four and twelve months after hospital discharge. The interventions included one-week wrist actigraphy and a home sleep apnea test (HSAT), and evaluations were conducted according to the Pittsburgh sleep quality index (PSQI), Epworth sleepiness scale (ESS), and insomnia severity index (ISI). Fifty-two patients were evaluated (ARDS = 31 and non-ARDS = 21); they had a median age of 49.0 [39.0;57.2] years and 53.8% were male. After twelve months, 91.3% presented poor sleep quality, 58.7% presented insomnia, 50% presented daytime somnolence, and 37% presented comorbid insomnia and obstructive sleep apnea (COMISA). No significant improvement was observed in relation to sleep or the circadian rest–activity rhythm between four and twelve months. A tendency of poor sleep quality, insomnia, daytime somnolence, and COMISA was observed. Finally, there was no significant impact on the circadian rest–activity rhythm between four and twelve months or between the groups.

## 1. Introduction

Due to the COVID-19 pandemic, an increase in ICU occupations throughout the global population was observed. In this context, previous studies determined that at least 5% of the population may develop acute respiratory distress syndrome, which may lead to respiratory failure and, consequently, to the use of an IMV [1]. Moreover, cardiovascular, renal, hematological, and cutaneous manifestations have been reported [2,3,4,5,6]. 

According to recent data, the long-term sequelae after discharge from the ICU are unknown. However, recent research highlights that COVID-19 patients who were hospitalized experience persistent symptoms after medical discharge [7]. After medical discharge, several studies suggest that patients with ARDS have sleep and circadian rhythm alterations in the short and mid-term, such as insomnia, poor sleep quality, fragmentation of the rest–activity rhythm, and obstructive sleep apnea (OSA) [8,9,10,11,12]. In fact, moderate and severe OSA has been reported to be highly prevalent in survivors of ARDS due to SARS-CoV-2 infection (COVID-19) [13]. Previously, several studies have suggested higher spectral symptoms after acute infection in COVID-19 survivors, in which prolonged symptoms after acute infection are observed; this has been named post-acute COVID syndrome (PACS) [14,15,16,17,18,19]. Along the same line, current research has suggested an association between PACS and detrimental sleep health, neurologic alterations, respiratory consequences, and impaired quality of life [9,11,19,20,21]. This set of symptoms after the acute phase have been reported in both ARDS and non-ARDS patients. However, several studies agree that patients who developed ARDS during the acute phase of COVID-19 have a higher risk of developing symptoms during PACS [22]. The aim of this study was to evaluate the impact on the sleep and circadian rest–activity rhythm of ARDS patients among COVID-19 survivors within twelve months after hospital discharge.

## 2. Materials and Methods

We performed a prospective, observational cohort study in accordance with current guidelines for reporting observational studies [23]. This study included two hospitals in Chile (the Hospital Regional Dr. Guillermo Grant Benavente and the Complejo Asistencial Dr. Víctor Ríos Ruiz). The data extraction was conducted during two clinical visits at four and twelve months after medical discharge. We included patients >18 years old who had positive real-time reverse transcription polymerase chain reaction (rRT-PCR) tests for SARS-CoV-2 between April 2020 and July 2020. Our study design included two groups, according to World Health Organization recommendations [24]: (i) The ARDS group consisted of patients who required mechanical ventilation during their ICU stay due to the severity of their symptoms; (ii) the non-ARDS group was made up of patients who had COVID-19 with mild and moderate symptoms but who did not develop ARDS during the acute phase and who did not require hospitalization. We excluded patients with one or more of the following characteristics: (i) older than 70 years; (ii) had previous respiratory comorbidities; (iii) had oxygen supplementation or non-invasive mechanical ventilation after COVID-19 hospitalization; (iv) severe mental and/or physical disabilities that could prevent the proposed evaluations.

The study protocol was approved by the institutional review boards (IRBs) of Servicio de Salud Bio-Bio (IRB: CEC113) and Servicio de Salud Concepcion (IRB: CEC-SSC: 20-07-26) in Chile, signed informed consent was acquired prior to inclusion of the patients in the study, and all methods followed the Helsinki Declaration and good clinical practice.

### 2.1. Baseline Characteristics

At baseline, we extracted the following data: age, sex, comorbidities, years of education, place of residence, tobacco use, alcohol use. Additionally, we calculated the body mass index (BMI) (kg/m^2^) and collected the neck circumference (cm) of patients.

### 2.2. Sleep Questionnaires

All the participants completed the Spanish versions of the following questionnaires. 

(i) The Pittsburg sleep quality index (PSQI): This questionnaire is composed of nineteen questions. The first four are open questions, while questions five to nineteen are rated on a four-point Likert scale. The PSQI has a range of 0–21 points, with 0 indicating the absence of difficulties and 21 points indicating severe difficulties. Sleep quality was categorized as “good-quality sleep” (≤5 points) or “poor-quality sleep” (>5 points) [25,26].

(ii) Epworth Sleepiness Scale (ESS): This is a self-administered eight-item questionnaire to determinate excessive daytime somnolence. The total score was categorized as daytime sleepiness (>10) or no daytime sleepiness (≤10) [25].

(iii) Insomnia Severity Index (ISI): This is a questionnaire that assesses the nature, severity, and impact of insomnia. It is made up of seven questions that evaluate the following dimensions: severity of sleep onset, sleep maintenance and early morning awakening problems, sleep dissatisfaction, interference of sleep difficulties with daytime functioning, perception of problems of sleep by others, and distress caused by sleep difficulties. A five-point Likert scale is used to rate each item, yielding a total score ranging from 0–28. The total score was interpreted as follows: no insomnia (0–7); insomnia below threshold (8–14); moderate insomnia (15–21); or severe insomnia (22–28). A cutoff of >7 points was used to indicate insomnia [26].

### 2.3. Home Sleep Apnea Test (HSAT)

The presence of sleep apnea was evaluated through a home sleep apnea test (HSAT) (Apnea Link Air, ResMed, Sydney, Australia), which was performed according to the American Academy of Sleep Medicine (AASM) recommendations [27]. OSA was defined by an RDI ≥ 5 ev/h, and moderate to severe OSA was defined by an RDI ≥ 15 ev/h [27]. Additionally, the combination of comorbid obstructive sleep apnea and insomnia (COMISA) was identified using the following parameters: RDI ≥ 5 ev/h + ISI > 7 points. 

### 2.4. Actigraphy

We performed a seven-day wrist actigraphy test (ActTrust 2-AT20101 Wrist accelerometer, São Paulo, Brazil) while keeping a daily diary. We examined the following parameters: time in bed (TIB) (min), total sleep time (TST) (min), and sleep efficiency (SE) (%) [28]. Additionally, to describe the 24 h rest–activity rhythm, we extracted the following activity data from the actigraphy: interdaily stability (IS), which ranges from 0–1 and indicates the synchronization between endogenous rhythms and external cues; intradaily variability (IV), which ranges from 0–2 and indicates the fragmentation of the rest–activity rhythm within each 24 h period; and the circadian function index (CFI) which ranges from 0–1 and is calculated as the average between the IS, IV, and RA [29]. Additionally, we considered the relative amplitude (RA), which ranges from 0–1, representing the differences in the magnitude of activity between active and rest phases (M10 − L5/M10 + L5), the most active ten-hour period (M10), and the least active five-hour period (L5).

### 2.5. Statistical Analysis

Descriptive statistics of the median (25th percentile; 75th percentile) were estimated for quantitative variables. The absolute and relative frequencies were calculated for qualitative variables. Relative frequencies were calculated excluding missing data. Comparisons of baseline characteristics between the study groups (ARDS and non-ARDS) were accomplished with the Wilcoxon signed-rank test for continuous variables and a chi-squared test (or Fisher exact test when the expected frequencies were less than five in some cells) for qualitative variables. 

Comparisons of sleep and circadian-related parameters between the study groups (ARDS and non-ARDS) at the twelve-month follow-up were performed with unadjusted and adjusted logistic or linear models (depending on the characteristics of the outcome). Age, sex, and BMI were considered as confounding factors in the adjusted models. Changes in sleep and circadian-related parameters over time (between the four- and twelve-month follow-up), stratified by the study groups (ARDS and non-ARDS), were assessed using generalized linear mixed-effect models adjusted for confounding factors (age, sex, and BMI), including the patient as a random effect. The differences between the groups of study (ARDS and non-ARDS) at each time point (four- and twelve-month follow-up) were assessed for interaction between the group and time. The *p*-value threshold defining statistical significance in all the analyses was set at 0.05. Data management and statistical analyses were performed using R (version 4.0.2; R Foundation for Statistical Computing).

## 3. Results

### 3.1. Baseline Characteristics Four Months after Medical Discharge 

The global cohort was composed of 52 patients (non-ARDS = 21 and ARDS = 31); they had a median [p25;p75] age of 49.0 [39.0;57.2] years and 53.8% were male (Table 1). The most prevalent comorbidity was hypertension (38.5% of patients). The ARDS patients were older compared to non-ARDS patients (54.0 [45.5;59.0] years vs. 41.0 [34.0;49.0] years, *p*-value = 0.010), had a higher prevalence of insulin resistance (10 (32.3%) vs. 1 (4.76%) *p*-value = 0.034), and exhibited significative differences (*p* < 0.05) related to the age of 54.0 [45.5;59.0]. Additionally, 54.8% of the ARDS group had less than eight years of schooling, and they had a significantly high prevalence of insulin resistance (32.3%) (*p*-value 0.03) (Table 1).

### 3.2. Sleep and Circadian Rest–Activity Rhythm 12 Months after Hospital Discharge

Regarding sleep quality, 91.3% of the global population presented poor sleep quality twelve months after hospital discharge, while 50.0% presented daytime sleepiness (Table 2). Moreover, 58.7% of the patients reported insomnia. No differences were observed between ARDS and non-ARDS patients.

Based on actigraphy data, the TST presented a mean (SD) of 428 (±92.1) min whereas the SE exhibited a mean of 87.4% (±5.15). No differences were observed between ARDS and non-ARDS patients.

**Figure 1:**
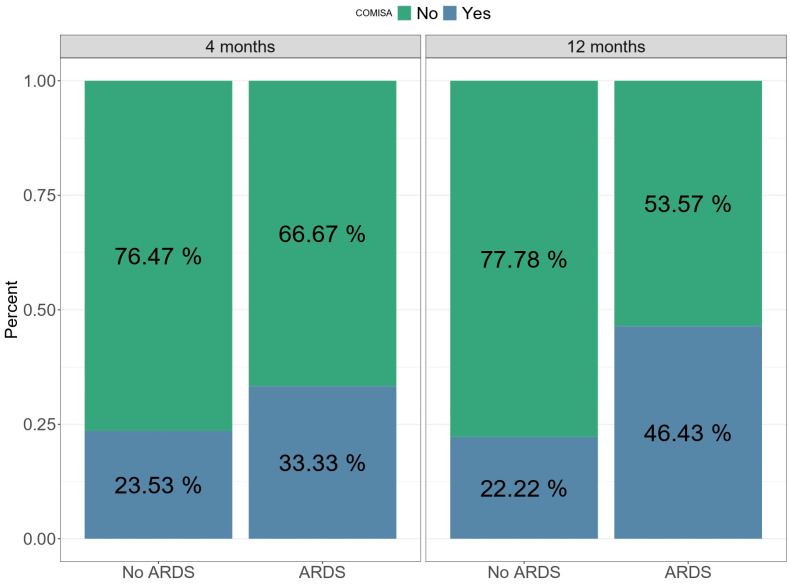
This figure shows the prevalence of COMISA between four and twelve months after medical discharge and the prevalence of COMISA in the non-ARDS and ARDS groups.

Regarding sleep apnea, the RDI presented a mean of 14.7 (±15.8) twelve months after medical discharge, while 37% of the global cohort presented COMISA. Only RDI showed significant differences between study groups, but the difference was not maintained after adjusting for confounding factors. Additionally, the COMISA variable was highly prevalent in the ARDS groups (46.4%) compared to the non-ARDS group (22.2%) (Figure 1), although no statistically significant differences were observed. 

### 3.3. Evolution of Sleep and Circadian Rest–Activity Rhythm between Four and Twelve Months after Hospital Discharge

**Figure 2:**
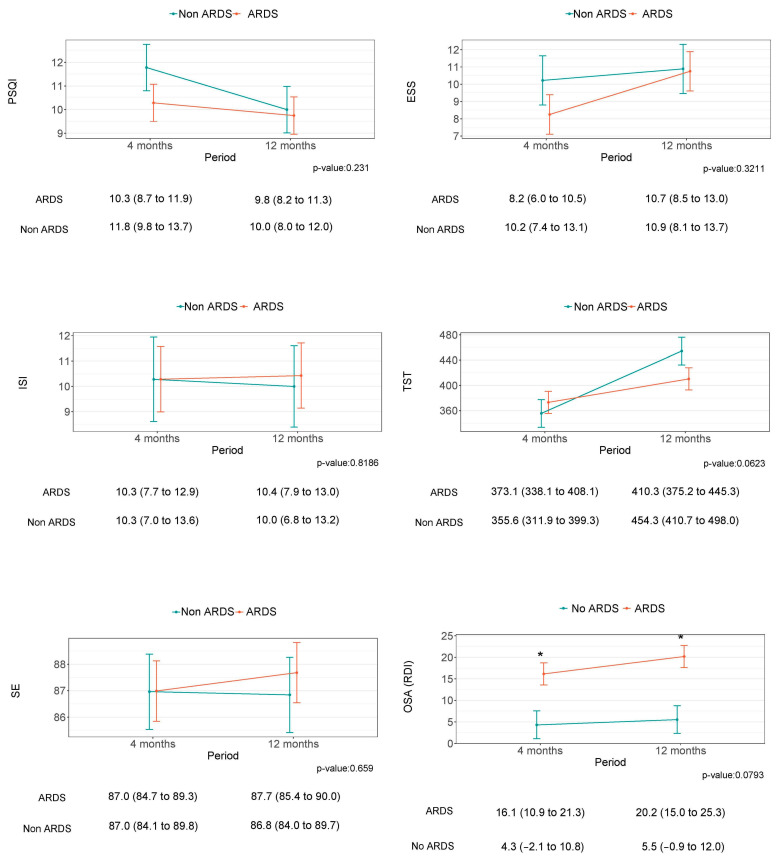
This figure shows the sleep differences between four and twelve months after medical discharge and the differences between the non-ARDS and ARDS groups. The difference between the groups is indicated by the ''*'' symbol.

**Figure 3:**
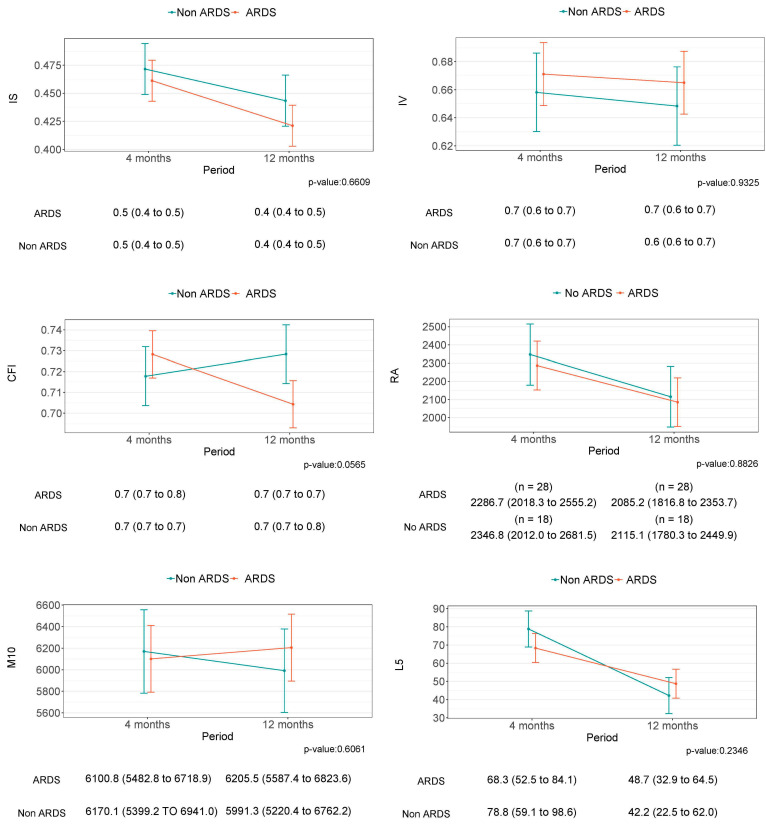
This figure shows the circadian rest–activity rhythm differences between four and twelve months after medical discharge and the differences between the non-ARDS and ARDS groups.

Despite some tendencies, especially in relation to the TST, there was no statistically significant improvement in any of the parameters describing sleep and circadian health between four and twelve months after hospital discharge (Figure 2 and Figure 3). Similarly, no differences were observed between the groups of study. 

## 4. Discussion

In the current study, we observed that 91.3% of COVID-19 survivors presented poor sleep quality twelve months after hospital discharge, in addition to a high prevalence of insomnia (58.7%) and daytime somnolence (50%). Additionally, 37% of the patients presented COMISA. Nevertheless, despite the high prevalence of sleep alterations among our population, no impact of ARDS was observed in either sleep or circadian health. Furthermore, the additional analysis did not show any significant improvement in sleep or the circadian rest–activity rhythm between the evaluated time points. 

Several studies describe a broad spectrum of sleep alterations in critical survivors. Delirium is common in critically ill adults [30], characterized by cognitive impairment and worse health outcome associations, with extensive strategies needed for pharmacological and non-pharmacological treatment [31,32]. In COVID-19-hospitalized patients, delirium was common, including in younger patients [33]. Indeed, the two conspicuous manifestations of circadian disruption in critical survivors are delirium and sleep disturbance [34].

Sleep in the studied population was characterized by architectural sleep alterations, sleep fragmentation, insomnia, poor sleep quality, and disordered breathing [21,35,36,37]. Sleep disturbance is common in ARDS survivors, being found up to one year after ICU discharge [36]. One of the most common sleep disturbances in this population is insomnia. In fact, insomnia is the most prevalent sleep disturbance one year after medical discharge from ICU [38]. Poor sleep quality in ARDS survivors was previously reported at three and six months after ICU discharge by Benitez and Targa, respectively [10,39]. Even though compromised sleep quality is to be expected in these survivors, the existing evidence after twelve months is scant in this area. Regarding sleep quality improvement, several studies have suggested better results as the number of months after discharge from the ICU increases [40,41]. In contrast, our results showed a high prevalence of poor sleep quality, which converges in the same direction as Altman’s systematic review [36]. 

Although our study found that ARDS survivors had higher RDI values twelve months after medical discharge, the difference was not maintained after adjusting for confounding factors. As it is known, SDBs such as OSA are considered a risk factor for developing severe COVID-19 [42]. Goyal A et al. (2021), concluded that OSA was highly prevalent in ARDS survivors due to COVID-19, and suggests that OSA might be an independent risk factor for poor prognosis in patients with COVID-19 [13]. According to González J et al. (2021), pulmonary structural abnormalities were highly prevalent in patients who developed ARDS due to COVID-19, and they showed an association between the severity of lung damage and the length of IMV during the ICU stay [20]. Moreover, our results revealed a high prevalence of COMISA at twelve months after medical discharge, with a higher prevalence in the ARDS group. According to Sweetman A (2019), this binomial consists of the co-occurrence of OSA and insomnia (COMISA). Indeed, 30–50% of patients with obstructive sleep apnea (OSA) report insomnia symptoms, whereas 30–40% of patients with insomnia have comorbid OSA [43], consequently triggering excessive daytime sleepiness, as observed in our results. The coexistence of these sleep alterations, in addition to physical disabilities [44], eating routine alteration, social behavior restriction [45,46,47,48,49,50], and neurocognitive alterations, could, in turn, lead to circadian clock dysregulation [51]. At the same time, they could potentiate the long-term alteration of sleep health and circadian rest–activity rhythms in ARDS survivors. 

Previous studies reported a high burden of symptoms and complications after acute infection among COVID-19 survivors, named post-acute COVID syndrome (PACS). PACS has been associated with an increased risk of insomnia and impaired quality of life [15,16]. Current evidence suggests expectable sleep alterations in ARDS survivors due to COVID-19, showing a prevalence up to 45% among survivors of severe COVID-19 infections [22]. However, and at the same time, studies have described an improvement related to sleep quality; for instance Targa et al., observed a slight sleep recovery between three and six months of follow-ups [39]. Additionally, a recent meta-analysis suggests that the persistence of symptoms was highly common in the long term (more than six months) compared to mid-term [14]. Currently, the evidence about changes in circadian rest–activity rhythms in ARDS survivors due to COVID-19 is scant and does not cover the alterations after one year from medical discharge. However, in previous studies in which the samples and tools of evaluation were similar to our research, a marked disturbance of the circadian rest–activity rhythm was found between three and four months after medical discharge [7,12]. 

According to our results no significant results were found to be related to the circadian rest–activity rhythm. However, we observed a persistent tendency to fragmentation and the least stability in the circadian rest–activity rhythm twelve months after medical discharge. The causality of alteration to circadian rest–activity rhythms is unknown and is beyond the scope of this study. However, the circadian rest–activity rhythm may be affected due to the regulatory effect of the circadian clock over physiological functions in the human body such as respiratory function, immune system behavioral aspects, and the metabolic system [52]. In turn, owing to massive infection by pathogenic entities such as SARS-COV-2, these physiological functions are exhibited with alterations after ICU discharge [20,53]. Indeed, Papagerakis et al. (2022) suggested potential connections between circadian rhythms, clock genes, and SARS-CoV-2 [52]. The additional analysis did not show any significant improvement in sleep and circadian rest–activity rhythm between the evaluated time points; such results may suggest that the effects of hospitalization and the acute phase of the disease are not the responsible for the sleep and circadian alterations. Possible reasons for the maintenance of such alterations are: (i) they were already present before the infection; (ii) they are the consequence of other sequelae such as mental health, physical capacity, respiratory function; in fact, the persistence of factors such as neuromuscular symptoms [54], irregular behavior and lifestyles [52], pulmonary structural abnormalities and functional impairment [20], and neurocognitive impairment have been widely described in ICU survivors [54]. All these factors, in addition to the loss of synchronization between the central and peripheral clock, could potentiate long-term alterations in the circadian rest–activity rhythm twelve months after medical discharge [52]. Moreover, several studies suggested that physical treatment and rehabilitation play a significative role in the recovery of long COVID-19 patients, improving several aspects such as physical function, pain, and pulmonary and cognitive function [55,56,57]. However, this intervention was not included in our study. 

To our knowledge, this is the first study that reports sleep health results between four and twelve months after medical discharge in COVID-19 survivors through actigraphy wrist tests, home sleep apnea tests, and validated questionnaires. We believe that these results can help visualize the impact on sleep health and the circadian rest–activity pattern due to COVID-19. Furthermore, these results can reinforce existing knowledge about the differences in sleep health and circadian rest–activity patterns between ARDS and non-ARDS patients, as well as knowledge about these symptoms or alterations in sleep health and circadian rest–activity patterns twelve months after medical discharge. Additionally, these results can collaborate with clinical aspects to help professionals understand that the manifestations and complications due to COVID-19 are more extensive than we imagine. This new health scenario must make health decision-makers reflect on the coverage that plans and programs should have for patients who have been discharged following COVID-19 infection and on the care and attention that we are providing to the survivors of this pandemic.

The main limitations of this study are: (i) the nest cohort, including a small sample size; (ii) we used subjective measures of different sleep-related parameters including sleep-related symptoms (ESS, PSQI, ISI); (iii) due to the COVID-19 pandemic, it was not feasible to perform a baseline evaluation of sleep and circadian function, which would have enabled the identification of possible sleep and circadian alterations prior to hospitalization or SARS-CoV-2 infection. Therefore, the study results should be interpreted with caution. Finally, although we provide more information about sleep health, (iv) the lack of polysomnography is also a limitation. Finally, through this research, we were not able to determine causality sequelae between the correlation of the variables of interest. Further studies exploring the contribution of this study are relevant to better understand sleep health.

## 5. Conclusions

Compared to non-ARDS survivors of COVID-19 in our sample, there is a high burden of sleep disturbances among ARDS survivors twelve months after hospital discharge. The rates of chronic insomnia, excessive daytime sleepiness, and COMISA are high after ARDS in the group studied. Finally, there was no significant impact on the circadian rest–activity rhythm between four and months or between the groups. 

## Figures and Tables

**Table 1:** Cohort baseline characteristics after medical discharge.

	Global	Non-ARDS	ARDS	
	*(N = 52)*	*(N = 21)*	*(N = 31)*	
	*Median [p25;p75] or n(%)*	*Median [p25;p75] or n(%)*	*Median [p25;p75] or n(%)*	*p-Value*
Baseline characteristics				
Age, years	49.0 [39.0;57.2]	41.0 [34.0;49.0]	54.0 [45.5;59.0]	0.010
Male	28 (53.8%)	7 (33.3%)	21 (67.7%)	0.031
Smoking history				0.094
*Non-smoker*	32 (61.5%)	14 (66.7%)	18 (58.1%)	
*Current*	8 (15.4%)	5 (23.8%)	3 (9.68%)	
*Former*	12 (23.1%)	2 (9.52%)	10 (32.3%)	
Alcohol				0.481
*No*	22 (42.3%)	8 (38.1%)	14 (45.2%)	
*Usually,*	28 (53.8%)	13 (61.9%)	15 (48.4%)	
*Frequent*	2 (3.85%)	0 (0.00%)	2 (6.45%)	
BMI	30.4 [28.0;33.5]	29.0 [27.9;32.0]	31.4 [28.7;35.4]	0.088
Neck circumference	41.5 [39.0;45.2]	39.0 [37.0;43.0]	42.0 [41.0;46.0]	0.011
Years of education				0.020
<8 years	21 (40.4%)	4 (19.0%)	17 (54.8%)	
8–12 years	10 (19.2%)	4 (19.0%)	6 (19.4%)	
>12 years	21 (40.4%)	13 (61.9%)	8 (25.8%)	
Place of residence, rural area	6 (11.5%)	1 (4.76%)	5 (16.1%)	0.382
Comorbidities				
Hypertension	20 (38.5%)	6 (28.6%)	14 (45.2%)	0.360
Diabetes mellitus,	7 (13.5%)	4 (19.0%)	3 (9.68%)	0.420
Insulin resistance	11 (21.2%)	1 (4.76%)	10 (32.3%)	0.034

Abbreviations: ARDS: acute respiratory distress syndrome; BMI: body mass index; SD: standard deviation; OR: odds ratio. *p* < 0.05 was considered significant for all analyses.

**Table 2:** Sleep and circadian health twelve months after hospital discharge.

	Global (N = 52) Mean ± SD or n (%)	Non-ARDS (N = 21) Mean ± SD or n (%)	ARDS (N = 31) Mean ± SD or n (%)	*N*	Unadjusted OR (95% CI) or Mean Difference (95%CI)	*p*-Value	Adjusted OR (95%CI) or Mean Difference (95%CI)	*p*-Value
Sleep								
PSQI	42 (91.3%) *	10.0 (3.34)	9.75 (4.64)	46	−0.25 (−2.73 to 2.23)	0.84	1.13 (−2.16 to 4.42)	0.50
ESS	23 (50.0%) *	10.9 (7.09)	10.8 (6.68)	46	−0.14 (−4.19 to 3.91)	0.94	1.30 (−4.09 to 6.69)	0.63
ISI	27 (58.7%) *	10.0 (5.64)	10.4 (7.74)	46	0.43 (−3.72 to 4.57)	0.84	2.51 (−3.06 to 8.08)	0.38
TST, (min)	428 ± 92.1	454 (69.7)	410 (101)	46	−44.08 (−97.69 to 9.53)	0.11	−55.01 (−128.09 to 18.06)	0.14
SE	87.4% (5.15)	86.8 (3.29)	87.7 (6.09)	46	0.84 (−2.23 to 3.92)	0.59	0.30 (−3.71 to 4.30)	0.88
Circadian rhythms								
IS	0.43 (0.11)	0.44 (0.10)	0.42 (0.11)	46	−0.02 (−0.09 to 0.04)	0.49	−0.06 (−0.14 to 0.02)	0.15
IV	0.66 (0.14)	0.65 (0.12)	0.66 (0.15)	46	0.02 (−0.06 to 0.10)	0.68	0.00 (−0.11 to 0.10)	0.95
CFI	0.71 (0.07)	0.73 (0.06)	0.70 (0.07)	46	−0.02 (−0.06 to 0.02)	0.23	−0.04 (−0.09 to 0.01)	0.15
RA	0.98 (0.02)	0.98 (0.01)	0.98 (0.02)	46	0.00 (−0.01 to 0.01)	0.54	0.00 (−0.01 to 0.02)	0.60
M10	6122 (1813)	5991 (1681)	6206 (1918)	46	214.22 (−869.34 to 1297.78)	0.70	−40.73 (−1486.69 to 1405.23)	0.95
L5	46.2 (44.4)	42.2 (38.1)	48.7 (48.6)	46	6.49 (−20.06 to 33.04)	0.63	−9.76 (−44.35 to 24.83)	0.58
Sleep Apnea								
RDI	14.7 (15.8)	5.57 (7.31)	20.2 (17.1)	45	14.61 (6.00 to 23.21)	0.00	1.83 (−8.02 to 11.69)	0.71
Comisa	17 (37.0%)	4 (22.2%)	13 (46.4%)	46	3.03 (0.80 to 11.54)	0.10	2.37 (0.43 to 13.06)	0.32

Abbreviations: *: abnormal prevalence; ARDS: acute respiratory distress syndrome; PSQI: Pittsburgh sleep quality index; ESS: Epworth sleepiness scale; ISI: Insomnia severity index; TST: total sleep time; SE: sleep efficiency; IS: interdaily stability; IV: intradaily variability; CFI: circadian function index; RA: relative amplitude; M10: most active ten-hour period; L5: least active five-hour period; RDI: respiratory disturbance index; COMISA: combination of comorbid obstructive sleep apnea and insomnia; SD: standard deviation; OR: odds ratio; CIs: confidence intervals. An adjusted *p* < 0.05 was considered for all analyses.

## Data Availability

Data will be shared by reasonable request. Contact the corresponding author.
